# Better restoration of joint line obliquity in tibia first restricted kinematic alignment versus mechanical alignment TKA

**DOI:** 10.1007/s00402-024-05551-8

**Published:** 2024-09-11

**Authors:** Ittai Shichman, Aidan Hadad, Addy S. Brandstetter, Itay Ashkenazi, Yaniv Warschwaski, Aviram Gold, Nimrod Snir

**Affiliations:** Adult Reconstruction Unit, Division of Orthopedic Surgery, Tel Aviv Sourasky Medical Center, NYU Langone Orthopedic Center, 6 Weizman St. 6th Floor, Tel-Aviv, Israel

**Keywords:** Total knee arthroplasty, Restricted inverse kinematic alignment, Mechanical alignment, Joint line obliquity, Restoration, Knee osteoarthritis

## Abstract

**Introduction:**

In total knee arthroplasty (TKA), suboptimal restoration of joint line obliquity (JLO) and joint line height (JLH) may lead to diminished implant longevity, increased risk of complications, and reduced patient reported outcomes. The primary objective of this study is to determine whether restricted kinematic alignment (rKA) leads to improved restoration of JLO and JLH compared to mechanical alignment (MA) in TKA.

**Materials and Methods:**

This retrospective study assessed patients who underwent single implant design TKA for primary osteoarthritis, either MA with manual instrumentation or rKA assisted with imageless navigation robotic arm TKA. Pre- and post-operative long standing AP X-ray imaging were used to measure JLO formed between the proximal tibial joint line and the floor. JLH was measured as the distance from the femoral articular surface to the adductor tubercle.

**Results:**

Overall, 200 patients (100 patients in each group) were included. Demographics between the two groups including age, sex, ASA, laterality, and BMI did not significantly differ. Distribution of KL osteoarthritis classification was similar between the groups. For the MA group, pre- to post-operative JLO significantly changed (2.94° vs. 2.31°, *p* = 0.004). No significant changes were found between pre- and post-operative JLH (40.6 mm vs. 40.6 mm, *p* = 0.89). For the rKA group, no significant changes were found between pre- and post-operative JLO (2.43° vs. 2.30°, *p* = 0.57). Additionally, no significant changes were found between pre- and post-operative JLH (41.2 mm vs. 42.4 mm, *p* = 0.17). Pre- to post-operative JLO alteration was five times higher in the MA group compared to the rKA group, although this comparison between groups did not reach statistical significance (*p* = 0.09).

**Conclusion:**

rKA-TKA results in high restoration accuracy of JLO and JLH, and demonstrates less pre- and post-operative JLO alteration compared to MA-TKA. With risen interest in joint line restoration accuracy with kinematic alignment, these findings suggest potential advantages compared to MA. Future investigation is needed to correlate between joint line restoration accuracy achieved by rKA and enhanced implant longevity, reduced risk of post-operative complications, and heightened patient satisfaction.

## Introduction

Total knee arthroplasty (TKA) is a commonly performed surgical procedure for patients with severe knee osteoarthritis, aiming to relieve pain, improve joint function, and enhance quality of life [1, 2]. Despite the success of TKA, achieving accurate implant placement and restoration of joint line obliquity (JLO) and joint line height (JLH) poses an ongoing challenge. Studies have demonstrated that suboptimal joint line restoration can lead to reduced functional outcomes and increased risk of complications [3]. Complications arising from inadequate joint line restoration include altered tibiofemoral kinematics, joint instability, compromised ligamentous balance, decreased range of motion, mid-flexion instability, and post-operative pain in the anterior aspect of the knee [3–6]. Poor restoration of JLO may require significant soft tissue release in order to achieve a balanced knee. This can result in increased surgical trauma and potential damage to the surrounding soft tissue sleeve, leading to prolonged recovery and decreased post-operative function [7, 8]. A systematic review by Popat et al. has demonstrated a statistically significant negative correlation between JLH alteration and post-operative Knee Society Scores, suggesting that inadequate restoration of JLH can result in poorer functional outcomes for patients following TKA [9]. These findings imply that restoration of JLO and JLH can be valuable indicators when assessing the effectiveness and anatomical recovery of the knee joint post-TKA procedures.

The traditional approach of mechanical alignment (MA), which aims for a completely neutral alignment of the knee, has faced criticism due to its standardized approach that overlooks individual anatomy and involves excessive release of soft tissues [10]. In response to this challenge, kinematic alignment (KA) has emerged as an alternative method designed to restore the inherent anatomical features unique to each patient’s limb. The fundamental principle of KA involves precisely matching implant thickness to the amount of bone and cartilage removed, thereby reinstating the diverse pre-arthritic orientation of the knee joint observed across the population [11, 12]. While KA is deemed suitable for primary osteoarthritis without severe deformity, determining the optimal range for positioning total knee components in kinematic alignment remains an ongoing challenge. Moreover, the distinct differences between an osteoarthritic knee and its pre-arthritic state add complexity to this consideration [10]. To address these intricacies, restrictive kinematic alignment (rKA) has been introduced as a middle-ground compromise between MA and KA, preventing extreme implant positions [10, 13, 14]. Introducing an innovative approach to patient-specific alignment (PSA), the concept of tibia first rKA seeks to equalize medial and lateral resections during tibial resurfacing first, while maintaining the natural obliquity of the tibial joint line. With this method flexion and extension gaps are balanced, potentially reducing the risk of excessive tibial resection and post-operative complications related to the tibia [14].

Multiple studies have examined JLO and JLH restoration in TKA utilizing MA, KA, and rKA. It has been reported that post-operative JLO and JLH after KA more closely resembled those of pre-operative knees compared to MA [15–19]. In addition, several studies showed that post-operative JLO and JLH following “femur first” rKA more closely resembled those of pre-operative knees compared to MA [20–22]. However, there is a research gap concerning the evaluation of JLO and JLH restoration when employing “tibia first” rKA as opposed to MA in TKA, particularly using the same implant design.

The primary objective of this study is to determine whether tibia first rKA leads to improved restoration of JLO and JLH compared to MA in TKA.

## Methods

### Study design

This retrospective study examined all patients over the age of 18 who underwent primary TKA using a single posterior stabilized implant between 11/2017 and 09/2023 at a single urban institution, which comprises a large academic medical center and a tertiary orthopedic specialty hospital. Patients were excluded if they underwent TKA for post-traumatic arthritis or oncologic lesions or lacked sufficient pre-operative and post-operative long-standing radiographs. As JLO and JLH inherently changes while realigning valgus knee deformity, all valgus knees defined by isolated lateral osteoarthritis or Hip-Knee-Angle (HKA) > 0° were excluded from this analysis.

### Baseline demographics and stratification

Baseline demographics were extracted from electronic medical records and included age, body mass index (BMI), sex, and American Society of Anesthesiologists (ASA) class. Pre-operative severity of osteoarthritis was assessed using the Kellgren-Lawrence classification (KL) of osteoarthritis [23].

### Pre-operative radiographic assessment

**Fig. 1:**
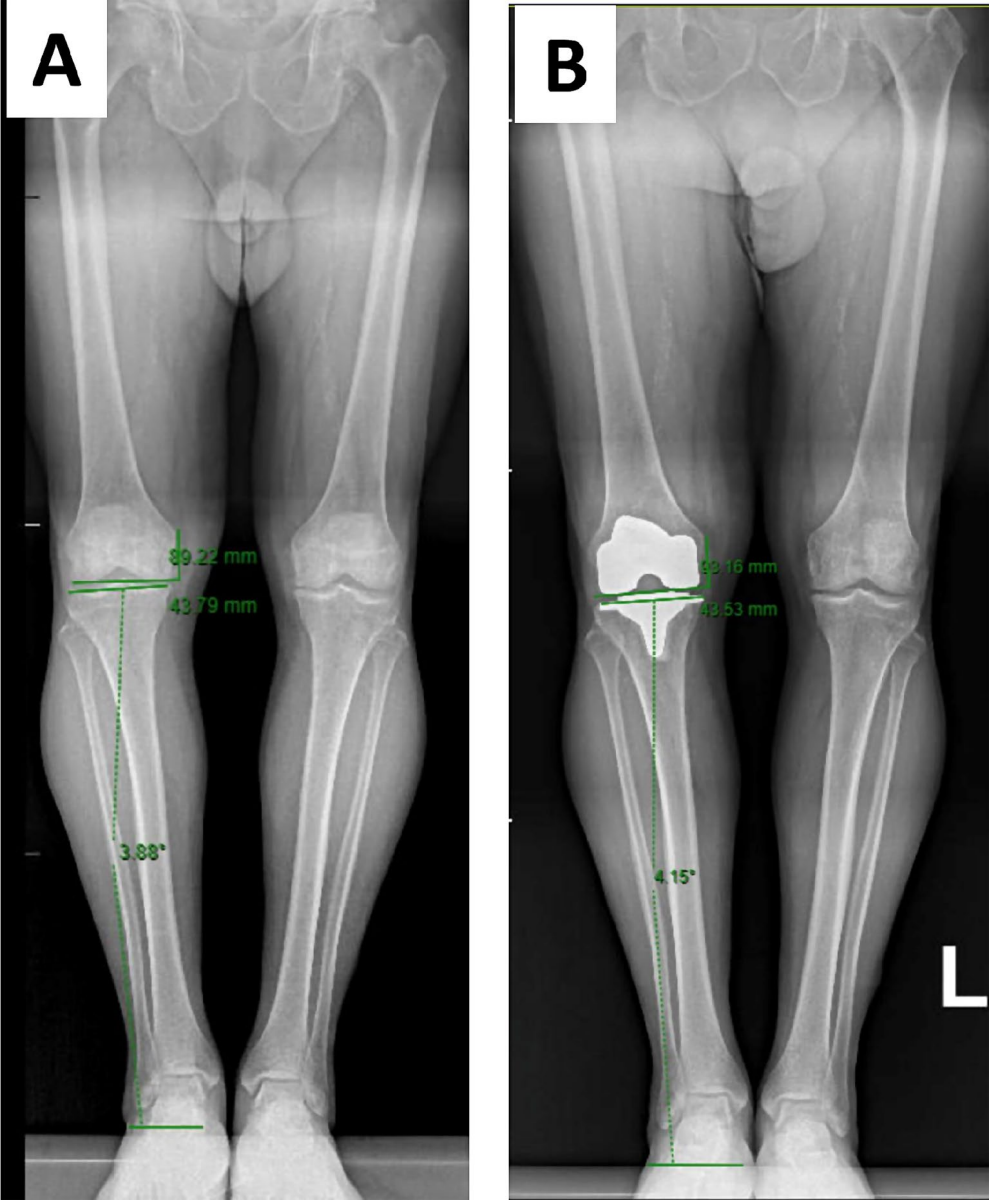
Preoperative **(A)** and postoperative (**B)** full-length standing AP radiographs with joint line obliquity and joint line height restoration measurements

Pre-operative and post-operative long standing AP X-ray imaging were used to measure JLO formed between the proximal tibial joint line and the floor. JLH restoration was measured as the distance from the femoral articular surface to the adductor tubercle (Fig. 1). Analysis was done on the Picture Archiving and Communication System (PACS) using the Visage 7 Imaging Software (Visage Imaging Inc, San Diego, CA, USA). All radiographs were reviewed by one of 2 fellowship-trained surgeons from the author group (IS and YW). Inter-observer reliability was tested using intra-class correlation coefficient (ICC) with a 2-way random effects model, assuming single measurements and absolute agreement. Sample size for reliability testing was calculated with an ICC target value of 0.8 and a 95% confidence interval width of 0.2. A minimum number of inter-observer reliability for the 2 raters was 20 by Bonnett’s approximation [24]. Thus, a subset of 20 radiographs were read by both the surgeons. ICC for measurements of JLO was 0.85 (0.79–0.90) and 0.83 (0.77–0.89) for JLH and was deemed excellent.

### Surgical data

All surgeries were performed by four adult reconstruction fellowship trained orthopedics with 7–20 years of experience post fellowship.

### Surgical technique

Patients were separated into two cohorts based on the utilized alignment philosophy:

1. Mechanical alignment (MA): which aims to achieve neutral mechanical axis passing from the center of the femoral head thorough the center of the knee joint line to the center of the ankle using manual instrumentation.
2. Tibia-first inverse kinematic alignment (iKA): This technique is a tibia first, gap balancing, with the aim to restore the native tibial joint line. Minimal adjustments are implemented on the femoral component position from the patient’s native femoral anatomy. Minimal releases if any are utilized in the goal of achieving a balanced knee in extension while maintaining the natural lateral laxity in flexion. In every instance, iKA was conducted utilizing imageless robot-assisted navigation employing a tibia-first approach, mirroring the technique outlined by Murgier et al. and Winnock de Grave et al. [14, 25]. A medial parapatellar approach with minimal medial release during exposure was employed. Subsequently, the navigation system was used to plan tibial resection, aiming to restore the native joint line in the coronal plane, while considering cartilage wear as described by Murgier et al. and restricting resection to 5° varus and 3° valgus from the mechanical axis [25]. Tibial resection was performed accordingly using the robotic cutting saw and validated using the navigation system validation pointer. A mechanical joint tensioner was then introduced into the joint space to collect laxity data through the range of motion prior to secondary balance assessment. The laxity data from the balance assessment were utilized as input for the intraoperative predictive gap-planning software, which virtually positioned the femoral component, providing a postoperative gap prediction throughout the range of motion. Femoral resections were planned to achieve stability and rectangular mediolateral gaps in extension, while permitting some lateral laxity as the knee transitions into flexion, restricting distal femoral valgus to 3° valgus and 6° varus from the mechanical axis using the predictive gap-planning software. Femoral resections were then carried out using the robotic cutting saw. Final laxity was determined using the implanted tibial insert thickness. Mediolateral balance was characterized as the disparity between lateral and medial laxity. All subsequent measurements were made intraoperatively and recorded into the robotic system user-interface.

Following anesthesia, the medial parapatellar approach was used in for all surgeries in both groups.

For the MA group surgeries, alignment was achieved by bone resections in the distal femur and proximal tibia perpendicular to the femoral and tibial mechanical axes in the coronal plane using intra and extramedullary rods and cutting jigs. To achieve appropriate extension and flexion gaps and patellar tracking, rotation of the femoral component, thickness of bone resection, and ligament balance were adjusted using the measured resection [26].

For the rKA group surgeries, the VELYS Robotic-Assisted imageless navigation system (Depuy-Synthes, Warsaw, IN) was used in all cases. Optical tracking arrays were mounted to the bones and anatomic landmarks were identified to construct anatomic coordinate systems. Surgeons planned their resection angles in real time using the system’s software. The target resection alignments were recorded, including the femoral sagittal angle, femoral coronal angle, femoral internal-external rotation angle, tibial sagittal angle, and tibial coronal angle, along with the thicknesses for the distal and posterior resections of the medial femoral condyle (femoral distal resection and femoral posterior resection) and tibial resection. ATTUNE (Depuy-Synthes, Warsaw, IN) posterior stabilized cemented knee system implants were used in all cases.

### Statistical analysis

Categorical variables were compared using chi-square tests or Fisher’s exact tests, and continuous variables were compared using independent-sample t-tests. Freedom from revision at latest follow-up was analyzed using the Kaplan-Meier method and compared with the log rank test. Significance was set at a p-value < 0.05. Categorical variables are represented as count, percentage, and continuous variables are represented as average ± standard deviation. Statistical analysis was done using R Statistical Software (version 2.4.1; 2022; R Foundation for Statistical Computing, Vienna, Austria).

## Results

Overall, 200 patients (100 patients in each group) were included. Demographics between the two groups including age, sex, ASA, laterality, and BMI did not significantly differ. Distribution of KL osteoarthritis classification was similar between the groups ((Table 1). For the MA group, pre- to post-operative JLO significantly changed (2.94° vs. 2.31°, *p* = 0.004). No significant changes were found between pre- and post-operative JLH (40.6 mm vs. 40.6 mm, *p* = 0.89). For the rKA group, no significant changes were found between pre- and post-operative JLO (2.43° vs. 2.30°, *p* = 0.57). Additionally, no significant changes were found between pre- and post-operative JLH (41.2 mm vs. 42.4 mm, *p* = 0.17) (Table 2). Pre- to post-operative JLO alteration was five times higher in the MA group compared to the rKA group, although this comparison between groups did not reach statistical significance (*p* = 0.09).

**Table 1:** Patient demographics

	MA (*n* = 100)	iKA (*n* = 100)	*P*-value
Mean Age, years (SD)	70.8 (8.9)	69.8 (9.1)	0.27
Female Sex, n (%)	75 (75)	69 (69)	0.34
Mean BMI, Kg/m^2^ (SD)	30.9 (5.1)	31.0 (5.7)	0.23
Laterality (right), n (%)	44 (44)	51 (51)	0.32
ASA Class, n (%)			0.37
1	4 (4)	6 (6)	
2	68 (68))	74 (74)	
3	28 (28)	20 (20)	
Kellgren Lawrence Grade, n (%)			0.23
2	26 (26)	17 (17)	
3	43 (43)	53 (53)	
4	31 (31)	30 (30)	

MA, Mechanical Alignment; iKA, Inverse Kinematic Alignment; SD, Standard Deviation; BMI, Body Mass Index; ASA, American Society of Anesthesiologists

**Table 2:** Changes in joint line obliquity and joint line hei

	Pre-op value	Post-op value	*P*-value
**MA Group (** ***n = 101)***			
Mean JLO, Degrees (SD)	2.94 (1.9)	2.31 (1.5)	0.004
Mean JLH, mm (SD)	40.6 (4.5)	40.6 (4.8)	0.89
**iKA Group (** ***n = 101)***			
Mean JLO, Degrees (SD)	2.43 (1.8)	2.30 (1.4)	0.57
Mean JLH, mm (SD)	41.2 (6.6)	42.4 (6.2)	0.17

MA, Mechanical Alignment; iKA, Inverse Kinematic Alignment; JLO, Joint Line Obliquity; JLH, Joint Line Height; SD, Standard Deviation

## Discussion

The key findings of this study were that (1) significant alteration in JLO was observed in pre- and post-operative MA-TKA, while no significant JLO alteration was demonstrated in rKA-TKA (2) neither MA-TKA nor rKA-TKA showed significant changes in JLH before and after surgery (3) rKA-TKA showed a non-significant trend of better restoration of JLO compared to MA-TKA.

To our knowledge, this study represents the first comprehensive evaluation of both JLO and JLH restoration in “tibia first” rKA-TKA compared to MA-TKA, using a single implant design.

Our study revealed significant pre- and post-operative JLO alteration when using MA. Corban et al. reported a mean post-operative JLO alteration of 5.8° ± 3.5 in their study involving MA [27]. Another study by Ji et al. found a mean post-operative JLO alteration of 1.0° ± 2.2 with MA [15]. Compared to the aforementioned studies, the JLO alteration observed in our MA group is smaller. This finding can be explained by the exclusion of valgus knees. Using MA for realigning valgus deformity would in most cases change the JLO to neutral.

Our study demonstrated adequate pre- to post-operative JLH restoration in MA. Cozzarelli et al. reported a mean post-operative JLH alteration of 2.7 ± 1.9 mm in their study using MA [28]. Similarly, Richards et al. found a mean post-operative JLH alteration of 1.04 ± 0.39 mm with MA [20]. The observed JLH alteration in our MA group aligns closely with the findings reported in the existing literature to further validate the similarity of our MA cohort and its post operative joint line alteration.

Our study exhibited high accuracy of post-operative JLO restoration when using Tibia First rKA. Grave et al. found a mean JLO alteration of 1.3° ± 1.2 with riKA [14]. Another study by Orsi et al. reported a mean JLO alteration of 2.5° ± 3 when employing rKA (femur first reconstruction) [29]. The pre- and post-operative JLO results from our study using rKA exhibit a lesser degree of alteration compared to the JLO alteration reported in the literature. Once again, the cohorts of the limited literature available included both varus and valgus knee deformities which could explain the differnces found in JLO restoration in our study. Importantly, our study suggests that starting with planned tibial resections and then adjusting the femur preferentially (“tibia first”) restores equivalent JLO to the constitutional pre-operative JLO. This was not the case when MA initial plans were used, as the joint line became more neutral.

Our study demonstrated precise post-operative JLH restoration in riKA. Richards et al. reported a mean post-operative JLH alteration of 1.05 ± 0.41 mm in their study using rKA (femur first reconstruction) [20]. Similarly, Tuecking et al. found a mean post-operative JLH alteration of 1.6 ± 1.1 mm in their study using rKA (femur first reconstruction) [30]. The observed JLH alteration in our riKA group closely corresponds with the findings reported in the existing literature for similar alignment techniques. Notably, our study is the first to evaluate JLH alteration in rKA using “tibia first” reconstruction.

Limitations of this study include its retrospective design, which introduces selection bias inherent in data reliance on medical records. Furthermore, the small sample size reduces statistical power and limits the generalizability of findings, increasing the risk of type II errors. Importantly, this study focused on joint line restoration without clinical concordance in terms of stability and patient-reported outcomes. Long-term follow-up on patient-reported outcomes is necessary to identify a correlation between joint line restoration and patient satisfaction rates.

## Conclusion

In conclusion, Tibia first rKA-TKA results in restoration of JLO and JLH, and demonstrates less pre- and post-operative JLO alteration compared to MA-TKA. While rKA has been introduced as a middle-ground compromise between MA and KA, this study’s findings suggest high accuracy of post-operative restoration which closely resemble those of KA. Additional research is warranted to correlate between joint line restoration accuracy achieved by rKA and long-term clinical outcomes such as enhanced implant longevity, reduced risk of post-operative complications, and heightened patient satisfaction compared to conventional alignment techniques.
